# Cardiac T1 mapping in non-ST-segment elevation myocardial infarction: temporal changes in myocardial fibrosis

**DOI:** 10.3389/fcvm.2025.1563368

**Published:** 2025-05-23

**Authors:** Luis Paiva, Maria João Ferreira, Sónia Afonso, Paulo Donato, Lino Gonçalves

**Affiliations:** ^1^ Centro Académico e Clínico de Coimbra (CACC), Coimbra, Portugal; ^2^ Coimbra Institute for Clinical and Biomedical Research (iCBR), Universidade de Coimbra, Coimbra, Portugal; ^3^ Coimbra Institute for Biomedical Imaging and Translational Research, Universidade de Coimbra, Coimbra, Portugal; ^4^ Instituto de Ciências Nucleares Aplicadas à Saúde, Universidade de Coimbra, Coimbra, Portugal

**Keywords:** non-ST-elevation myocardial infarction, cardiac magnetic resonance, mapping, follow-up, microcirculation, late gadolinium enhancement, extracellular volume fraction

## Abstract

**Introduction:**

Cardiac magnetic resonance (CMR) imaging allows tracking of ongoing fibrosis modifications following myocardial infarction (MI). We evaluated temporal changes in late gadolinium enhancement (LGE) and extracellular volume fraction (ECV) within the MI culprit coronary artery and remote regions of the myocardium during the index ischemic event and follow-up in patients with NSTEMI.

**Methods:**

This prospective, single-center study included 30 patients with type 1 NSTEMI. It involved the evaluation of patients using coronary angiography, invasive coronary physiology, and biomarkers. CMR imaging was used to assess left ventricular (LV) volume, function, and myocardial fibrosis using LGE and ECV. These assessments were performed at baseline and repeated 6-10 months after MI.

**Results:**

At the 4-year post-MI follow-up, 27 patients survived [age 65 (58,74) years; 77% male], and LV mass, volume, and contractility remained unchanged between the baseline and follow-up measurements. Myocardial fibrosis assessed using LGE showed a decreasing trend at follow-up (9.4 ± 4.4% vs. 6.7 ± 4.4%; *p* = 0.051), particularly in the MI culprit coronary artery regions (14.2 ± 8.6% vs. 9.5 ± 7.0%; *p* = 0.015). LGE volume regression was observed in 70% of cases. The ECV measurements did not change between the initial and follow-up CMR assessments. Despite the high prevalence of multivessel coronary artery disease (CAD) (53%), no significant changes in LGE or ECV measurements were observed in the remote myocardium.

**Conclusions:**

After NSTEMI, LGE decreased in the heart regions supplied by the culprit coronary arteries. However, the ECV measurements remained unchanged. Multivessel CAD was not associated with significant changes in myocardial fibrosis in the remote myocardium.

## Introduction

Myocardial infarction (MI) triggers a series of tissue modifications, including inflammation, microvascular obstruction, hemorrhage, necrosis, and ultimately, the replacement of the heart muscle with fibrosis (1). The extent and severity of these changes impact prognosis. Cardiac magnetic resonance (CMR) imaging enables the assessment of structural and functional abnormalities in the myocardium resulting from ischemia/reperfusion injury, and provides the opportunity to track ongoing changes in the cell and matrix compartment following MI (2).

A distinctive feature of CMR is its ability to characterize tissue by utilizing proton relaxation times, such as T1, which can be employed to measure macromolecular content, water binding, and water content (3). When T1 is determined on a pixel-by-pixel basis, the resulting T1 values can be displayed as a T1 map. Furthermore, there has been a growing interest in using T1 weighted sequencing in the MI setting (4). CMR has become the standard method for quantifying both localized myocardial fibrosis, which can be measured through late gadolinium enhancement (LGE), and diffuse fibrosis, which is evaluated by calculating the extracellular volume fraction (ECV) (5).

Despite the current knowledge of patients with acute coronary syndrome, there is limited data regarding myocardial heart tissue characterization of non-ST-segment elevation MI (NSTEMI), particularly regarding the development of myocardial fibrosis. We aimed to assess changes in myocardial fibrosis over time using LGE and ECV within the MI culprit coronary artery heart regions and the remote myocardium. This cardiac imaging assessment was conducted at an early ischemic stage and during follow-up of patients with NSTEMI.

## Methods

### Study design and settings

**Figure 1:**
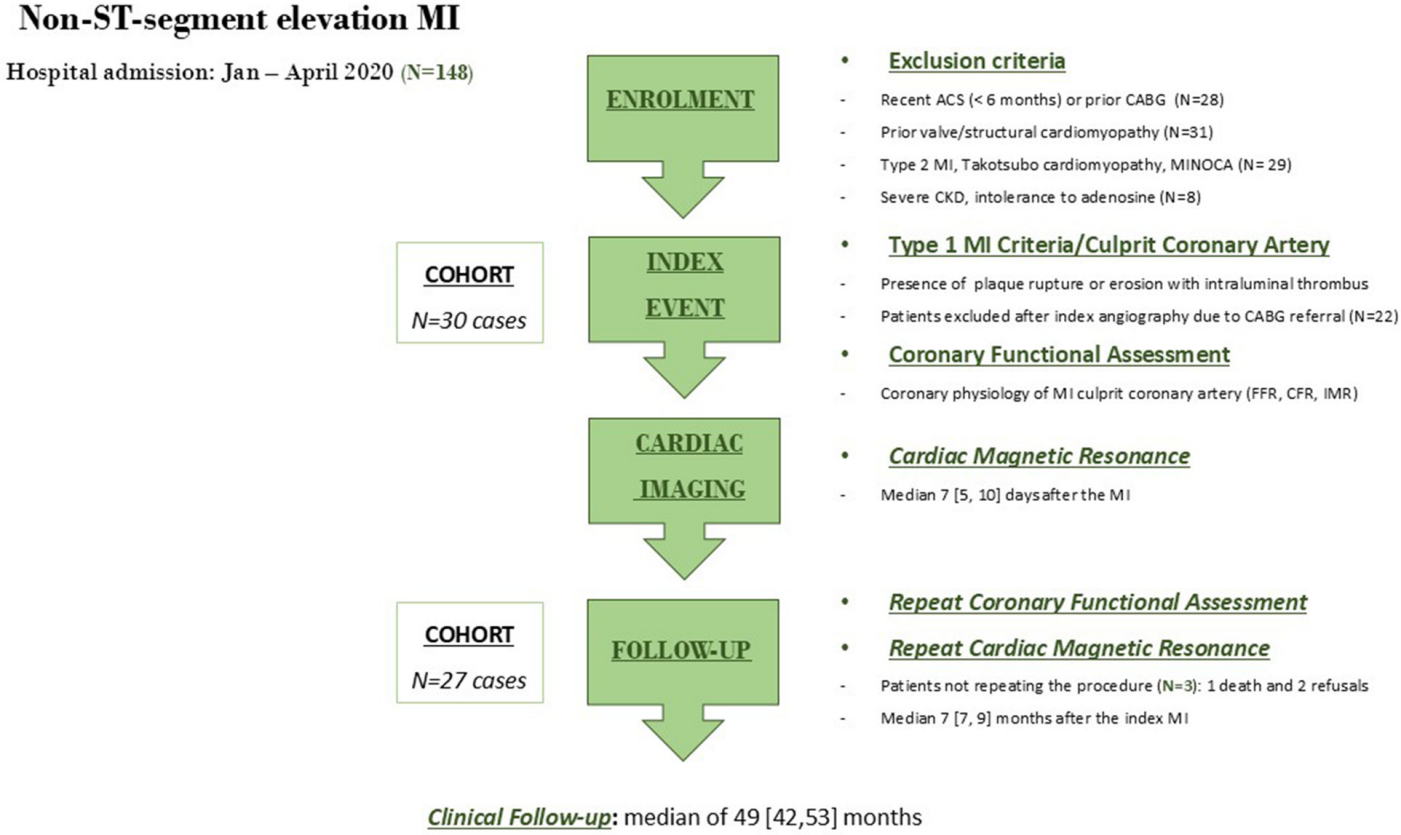
Flow diagram of the study. ACS, acute coronary syndrome; CABG, coronary artery bypass graft; CFR, coronary flow reserve; FFR, fractional flow reserve; IMR, index of microcirculatory resistance; MI, myocardial infarction; MINOCA, myocardial infarction with non-obstructive coronary arteries.

We conducted a prospective single-center study of 30 patients hospitalized for NSTEMI between January 1 and April 30, 2020. Patients were eligible for inclusion if they were over 18 years of age, had a clinical diagnosis of NSTEMI as defined by the universal criteria for MI (6) (patients with chest discomfort but without ST-segment elevation or left bundle branch block on ECG and elevated high-sensitivity cardiac troponin levels with at least one value above the 99th percentile of the upper reference limit), and provided written informed consent prior to enrollment. The key exclusion criteria were severe chronic kidney disease (glomerular filtration rate less than 30 ml/min/m^2^), significant concomitant valve disease or other structural cardiomyopathy, recent acute coronary syndrome (within the past 6 months), coronary artery anatomy considered for coronary artery bypass graft surgery, or ischemic myocardial injury resulting from a mismatch between oxygen supply and demand (type 2 myocardial infarction) (6). Patients who underwent coronary angiography at the index event were scheduled for repeat coronary angiography procedure within 6-10 months of the initial angiography (Figure 1). The MI culprit coronary artery was determined by the operator and later reviewed by an independent physician who considered data from 12-lead ECG, echocardiography, coronary angiography, and intracoronary imaging (6, 7). Coronary angiography findings of acute plaque rupture or acute vessel occlusion were considered key features for establishing type 1 MI and the culprit coronary artery. Additionally, patients were referred for a CMR study within 5–10 days of the index hospital discharge, which was then repeated 6–10 months after the first cardiac imaging study (Figure 1).

The data collected included demographic, anthropometric, clinical, laboratory, echocardiographic, angiographic, and CMR imaging characteristics. The study also collected follow-up information on overall mortality and major adverse cardiovascular events (MACE), defined as cardiovascular death, myocardial infarction readmission, and heart failure-related hospital admission. All data were anonymized prior to statistical analysis, and the study was conducted in accordance with the principles of the Declaration of Helsinki and approved by the local hospital ethics committee (CE-023/2017).

### Data collection

Upon hospital admission, blood samples were collected from each patient and analyzed using routine biochemical tests. The Ortho-Clinical Diagnostics VITROS® Troponin I ES Assay (Rochester, NY, USA) was used to measure troponin levels, with a 99th percentile for sensitivity and detection of 0.034 ng/ml and a lower limit of 0.012 ng/ml. Re-elevation of hsTn levels after percutaneous coronary intervention (PCI) was not considered an exclusion criterion in this study (8). Standard 12-lead ECGs were obtained upon admission and during hospital stay. The presence of abnormalities, such as ST-segment deviation, T-wave changes, Q-wave, and conduction and rhythm disturbances, was recorded. Routine transthoracic echocardiography was performed during the index hospital admission (median 2.7 days after admission) and throughout the follow-up period using a Vivid 7 ultrasound device (GE Healthcare, Horton, Norway). Echocardiographic studies with standard views were conducted according to established guidelines, and the left ventricular (LV) ejection fraction (EF) was calculated using Simpson's method (9). Clinical follow-up after hospital discharge was performed by reviewing the patients' hospital and general practitioner records, questionnaires, and the national registry of vital status.

### Angiographic procedure

Coronary angiography during the index hospital stay was a mandatory inclusion criterion for this study. Patients with significant obstructive CAD, defined as at least 70% diameter narrowing of a major coronary artery (or 50% narrowing in the left main coronary artery) (10) were considered for myocardial revascularization. The decision to pursue coronary surgery or PCI was based on current evidence, guidelines, and local clinical practices (7, 10). After the initial coronary angiography and PCI of the culprit vessel, a physiological assessment was conducted using the Coroventis CoroFlow Cardiovascular System (Abbott Laboratories, US) with the Abbott Pressure Wire™ X Guidewire under resting conditions and adenosine-mediated hyperemia at a weight-adjusted rate (140 μg/kg/min) by peripheral venous infusion (11). This assessment measured the fractional flow reserve (FFR), coronary flow reserve (CFR), and index of microcirculatory resistance (IMR) in the MI culprit and non-culprit coronary arteries. The design of our study required that patients undergo follow-up coronary angiography and physiological evaluation 6–9 months after the initial procedure. This approach aimed to assess the temporal changes in epicardial coronary disease and microvascular function during the follow-up period (Figure 1).

### CMR study

Patients underwent a CMR study with a 3.0-T CMR scanner (Magnetom Vida, Siemens Healthcare, Germany) using a stress/rest perfusion protocol (12, 13). Native myocardial T1 values were first obtained at rest using the established heart-rate-independent shortened look-locker inversion recovery (MOLLI) T1 mapping technique in three short-axis slice positions (basal, mid-ventricular, and apical). Myocardial perfusion imaging under stress conditions was performed 70s after intravenous administration of regadenoson (Rapiscan, GE Healthcare, US) at a fixed dose of 0.4 mg (5 ml) (14, 15). The vasodilatory effect of the drug was reversed with intravenous aminophylline (5 mg/kg) in all patients, regardless of the clinical symptoms. First-pass stress myocardial perfusion was conducted 150 s after regadenoson administration. Subsequently, 0.1 mmol/kg of gadobutrol was injected at a rate of 4.5 ml/s. LGE imaging was performed 10 min after gadolinium administration, and ECV was measured 15 min after contrast administration. The patients repeated the same CMR study protocol within 7–9 months of the initial cardiac imaging (Figure 1).

**Figure 2:**
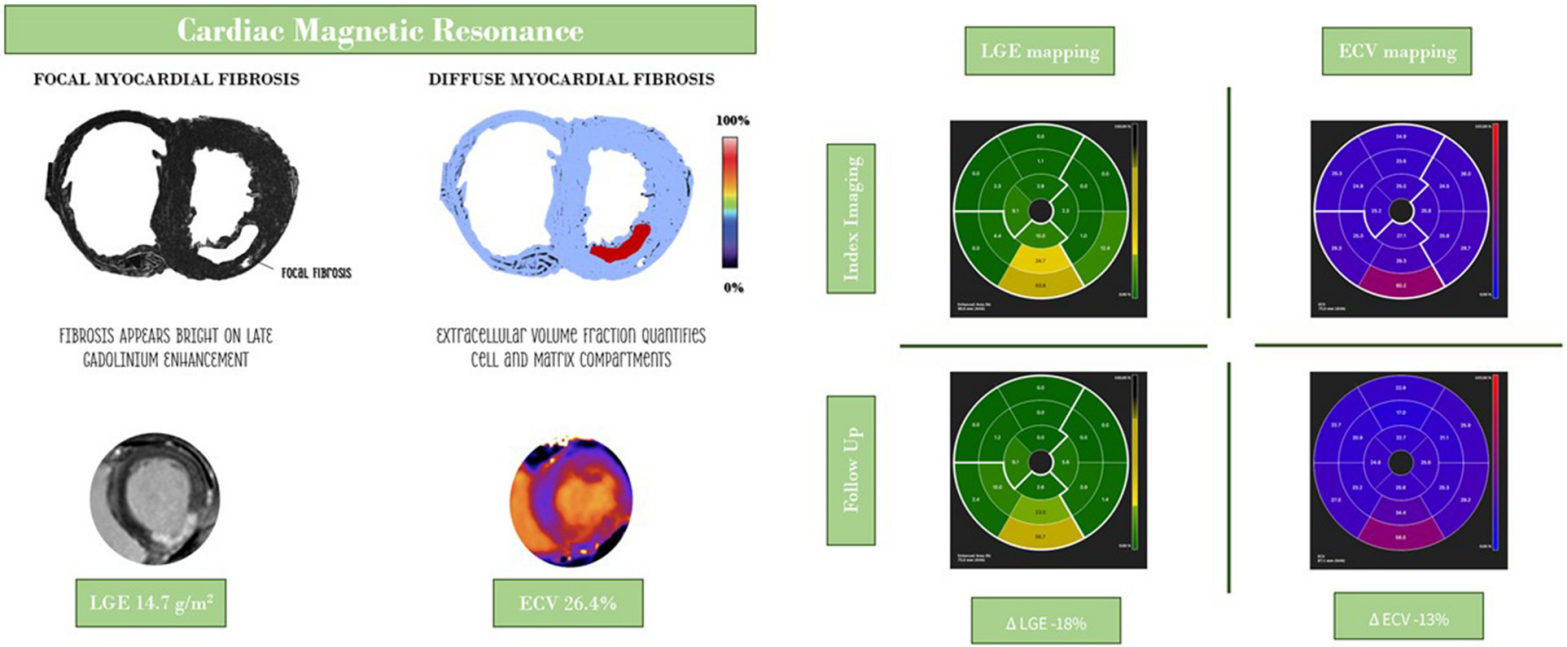
Central illustration: evaluation of localized and diffuse myocardial fibrosis following myocardial infarction. ECV, extracellular volume fraction; LGE late-enhancement gadolinium.

CMR studies were evaluated using specific software (CVI42®, Circle Cardiovascular Imaging, Canada). This analysis was initially conducted by a single observer (L.P.) and subsequently by a second independent observer (M. J. V.) who was blinded to the pathological or incidental findings (M.J.V.). The endocardial and epicardial contours were traced in the end-diastolic and end-systolic images to calculate the left ventricular volumes, function, and mass (3). The main quantitative myocardial fibrosis variables were native T1, LGE, and ECV (16–18). For LGE analysis, the mean signal intensity and standard deviation (SD) of the region of interest were measured, and enhanced myocardium was defined as myocardium with a signal intensity 5 SD above the remote normal myocardial signal. Diffuse myocardial fibrosis was quantified using ECV, defined as ECV = *λ*×(1- haematocrit), where *λ* = [ΔR1_myocardium_]/[ΔR1_bloodpool_] before and after gadolinium contrast (where R1 = 1/T1), with LGE regions included in the global and regional ECV assessment (16, 19). Haematocrit measurements were acquired on the day of scanning and measured in the clinical laboratory. The post-processing of CMR images involved analysing ventricular short-axis slices at the basal, middle, and apical levels. To evaluate the overall values, the measurements from all three sections were averaged. The heart was segmented according to the American Heart Association's 16-segment model and categorized by coronary artery territory, specifically distinguishing between MI culprit and non-culprit arteries regions. (Figure 2) In the quantitative analysis, regions of interest (ROI) were manually delineated within the mid-myocardial layer of each segment. These ROIs were placed to avoid the blood pool, epicardial fat, and myocardial borders, with an average size ranging from 80 to 120 mm^2^. In areas corresponding to the infarcted myocardial territory, the ROIs were positioned within segments exhibiting LGE to ensure spatial matching. For the remote myocardium, ROIs were selected in normokinetic, non-enhanced regions, devoid of wall motion abnormalities. Microvascular obstruction (MVO) was assessed using first-pass perfusion studies with LGE sequences (20).

A group of healthy individuals who underwent CMR study at 3.0-Tesla (*n* = 10) served as control subjects. These subjects underwent the same T1 mapping protocol as previously described for the NSTEMI group. The baseline clinical characteristics of the healthy controls are summarized in Table 1, and Table 3 provides information on LV volume, mass, and contractility.

**Table 1:** Baseline characteristics.

Cohort characteristics	Patients (*n* = 30)	Control (*n* = 10)	*p*-value
Male (%)	23 (77)	7 (70)	0.110
Age (years), median [IQR]	65 [58,74]	55 [46,65]	0.004
Arterial Hypertension (%)	22 (73)	0	–
Dyslipidaemia (%)	28 (93)	2 (20)	<0.001
Diabetes mellitus (%)	16 (53)	0	–
Smoker/Previous Smoker (%)	13 (43)	0	–
BMI (kg/m^2^), median [IQR]	28 [26,31]	29 [28,34]	0.884
GFR < 45 ml/min/1.73 m^2^] (%)	0	–	–
Previous MI/PCI (%)	5 (17)	–	–
Previous CABG (%)	0	–	–
Previous HF admission (%)	5 (17)	–	–
NTproBNP (pg/ml), median [IQR]	1,218 [342,1,921]	–	–
Peak hsTn (ng/ml), median [IQR]	7,121 [1,267–14,777]	–	–
ECG ST-segment deviation (%)	16 (53)	–	–
ECG negative T-wave (%)	11 (37)	–	–
GRACE_6m_ (score), median [IQR]	122 [108,139]	–	–
GRACE_6m_ death risk (%), median [IQR]	8 [5,14]	–	–
Echo LVEF, median [IQR]	57 [48,63]	59 [55,64]	0.533
One–vessel CAD	14 (47)	–	–
Two–vessel CAD	9 (30)	–	–
Three-vessel CAD	7 (23)	–	–
Medication at the index event			
Aspirin/P_2_Y_12_ receptor inhibitor (%)	25 (83)	–	–
Oral anticoagulant (%)	4 (13)	–	–
Statin (%)	28 (93)	–	–
ACE inhibitor/ARB/ARNI (%)	28 (93)	–	–
Beta-blocker (%)	23 (77)	–	–
Aldosterone Receptor Antagonist (%)	0	–	–
Medication during the first year of follow-up			
Aspirin/P_2_Y_12_ receptor inhibitor (%)	30 (100)	–	–
Oral anticoagulant (%)	4 (13)	–	–
Statin (%)	30 (100)	–	–
ACE inhibitor/ARB/ARNI (%)	29 (97)	–	–
Beta-blocker (%)	26 (87)	–	
Aldosterone Receptor Antagonist (%)	1 (3)	–	–

ACE, angiotensin-converting enzyme; ARB, angiotensin receptor blocker; ARNI, angiotensin receptor-neprilysin inhibitor; BMI, body mass index; CABG, coronary artery bypass grafting; CAD, obstructive coronary artery disease; CMR, cardiac magnetic resonance; ECG, 12-lead electrocardiogram; Echo Transthoracic echocardiography; GFR, glomerular filtration rate; HF, heart failure; hsTn, high-sensitivity cardiac troponin; IQR, interquartile range; LVEF, left ventricular ejection fraction; NTproBNP, N-terminal proB-type natriuretic peptide.

### Statistical analysis

Continuous variables are presented as mean ± standard deviation for normally distributed variables and as median values (25th-75th percentiles) for abnormally distributed variables. Categorical variables are presented as counts and proportions. The normality and homogeneity of the variances were tested using the Kolmogorov–Smirnov and Levene tests. Categorical variables were compared using χ^2^ or Fisher's exact test, and Student's *t*-test for normally distributed data or Mann–Whitney tests for non-normally distributed data. Paired Student's *t*-tests were used to evaluate the differences in continuous variables between the initial and subsequent measurements of native T1, LGE, and ECV. Statistical analyses were performed using SPSS version 22.0 (SPSS, Chicago, USA). *P-*values with a two-sided *α*-level of <0.05 were considered statistically significant.

## Results

### Study population

The baseline characteristics of the study cohort (*N* = 30) are shown in Table 1. The average hospital stay was 3.1 ± 0.9 days. The MI culprit coronary arteries were the LAD (*N* = 13, 43%), circumflex (*N* = 7, 23%), and right coronary artery (RCA) (*N* = 10, 33%). Multivessel obstructive CAD was identified in 16 (53%) patients in the study. Among those with multivessel obstructive CAD, eleven patients (69%) underwent non-culprit coronary artery revascularization. Chronic total occlusion (*N* = 1) or diffuse coronary artery disease/small-calibre coronary arteries (*N* = 4) were the primary reasons for not achieving complete myocardial revascularization at the index hospitalization.

### Clinical follow-up

All patients survived the initial hospitalization. The study followed the participants for a median period of 49 months [IQR 42,53]. Throughout the follow-up period, three patients (10%) died, one in the first 6 months (cardiovascular death) and the other after 42 months of follow-up (one cardiovascular and one non-cardiovascular death). During the follow-up coronary angiography conducted 6–9 months post-index event, no patients required subsequent percutaneous coronary intervention (PCI) for *de novo* lesions. However, one patient underwent PCI for chronic coronary occlusion 15 months following hospital admission, and another patient was referred for coronary artery bypass grafting (CABG) 14 months after the initial ischemic event due to recurrent angina associated with previous stent restenosis. Table 1 presents the use of medications during the first year of clinical follow-up in our cohort.

### Invasive physiological assessment of the coronary arteries at the index event and follow-up

The FFR, CFR, and IMR were assessed in 81 coronary arteries (culprit and non-culprit). The remaining nine coronary arteries could not be assessed because of chronic total occlusion (*N* = 1) and diffuse CAD/small-calibre coronary artery (*N* = 4) (operator discretion). Regarding the follow-up invasive procedures, one patient died before the next procedure, and two declined to undergo repeated coronary catheterization.

Concerning the culprit coronary artery, there was a significant improvement in the frequency of abnormal CFR values (<2.0) (60% vs. 27%, *p* = 0.013) and extensive microcirculatory dysfunction (IMR > 40) (17% vs. 4%, *p* = 0.03) at follow-up compared to measurements at the index angiographic procedure. The invasive functional assessment of MI non-culprit coronary arteries is presented in Table 2.

**Table 2:** Invasive functional assessment of the coronary arteries at the index ischemic event and follow-up procedure.

MI culprit coronary artery	Index event (*N* = 30)	Follow-up (*N* = 27)	*p*-value
Flow fractional reserve, median [IQR]	0.90 [0.84, 0.94]	0.86 [0.82, 0.98]	0.371
Coronary flow reserve, median [IQR]	1.8 [1.4, 2.7]	2.6 [1.8, 3.5]	0.102
Coronary flow reserve <2.0, N (%)	18 (60%)	7 (27%)	0.013
IMR, median [IQR]	20 [11, 32]	19 [12, 30]	0.242
IMR ≥25 and >40, N (%)	11 (37%), 5 (17%)	8 (31%), 1 (4%)	0.257, 0.030
Resistive reserve ratio, median [IQR]	2.3 [1.6, 3.2]	2.8 [1.8, 4.3]	0.149
MI non-culprit coronary arteries	Index event (*N* = 30)	Follow-up (*N* = 27)	*p*-value
Flow fractional reserve, median [IQR]	0.91 [0.83, 0.97]	0.91 [0.85, 0.95]	0.962
Coronary flow reserve, median [IQR]	2.1 [1.5, 3.8]	2.8 [2.3, 4.1]	0.127
Coronary flow reserve <2.0, N (%)	14 (47%)	5 (17%)	0.012
IMR, median [IQR]	18 [13, 31]	20 [14, 25]	0.167
IMR ≥25 and >40, N (%)	10 (33%), 1 (3%)	7 (26%), 1 (4%)	0.151, 0.312
Resistive reserve ratio, median [IQR]	3.1 [1.9, 4.8]	3.9 [2.7, 5.5]	0.095

IMR, index of microcirculatory resistance, IQR, interquartile range; MI, myocardial infarction.

### CMR findings at the index event and follow-up

The CMR study was conducted at a median of 7 [5, 10] days after the index ischemic event. During the follow-up period, most patients underwent repeat CMR imaging (*N* = 27, 90%). One patient died before reaching six months after the initial MI hospitalization, and two declined to undergo repeated cardiac imaging. Follow-up CMR was performed at a median of 7 months [IQR 7, 9] after the index hospitalization.

Table 3 presents the CMR data of patients with MI at baseline and during the follow-up period, showing no significant changes in the LV volume, mass, or ejection fraction over time. No significant rhythm disturbances, such as atrioventricular (AV) block or supraventricular/ventricular dysrhythmia, were observed during the initial or follow-up CMR procedures.

**Table 3:** Cardiac magnetic resonance data in myocardial infarction patients and control subjects.

CMR baseline characteristics	MI patients index event (*N* = 30)	MI patients follow-up (*N* = 27)	*p*-value	Control subjects (*N* = 10)	*p*-value^†^
Resting heart rate, beats/min	66 ± 11	64 ± 10	0.378	65 ± 10	0.315
Stress heart rate, beats/min	91 ± 10	90 ± 11	0.841	95 ± 11	0.278
Resting systolic blood pressure, mm Hg	138 ± 16	136 ± 18	0.732	134 ± 12	0.231
Stress systolic blood pressure, mm Hg	127 ± 18	129 ± 15	0.376	128 ± 14	0.355
Left ventricular ejection fraction, %	55 ± 12	57 ± 13	0.822	63 ± 7	0.443
LV end-diastolic volume/BSA, ml/m2	80 ± 15	84 ± 25	0.221	77 ± 8	0.134
LV end-systolic volume/BSA, ml/m2	37 ± 11	38 ± 22	0.320	32 ± 4	0.222
Left ventricular mass/BSA, g/m2	58 ± 12	58 ± 20	0.532	50 ± 8	0.038

BSA, body surface area; CMR, cardiac magnetic resonance; LV, left ventricle; MI, myocardial infarction.

^†^ MI index event compared to control subjects.

Native myocardial T1 did not significantly change between index and follow-up imaging (1,053 ± 52 ms vs. 1,026 ± 42 ms; *p* = 0.088). However, the native myocardial T1 decreased in the MI culprit coronary artery heart regions (1,089 ± 89 ms vs. 1,025 ± 62 ms; *p* = 0.013) during the follow-up period, whereas it remained unchanged in the MI remote areas. (Table 4) LGE showed a decreasing trend at follow-up (9.4 ± 4.4% vs. 6.7 ± 4.4%; *p* = 0.051), owing to an improvement in myocardial enhancement in the segments supplied by MI-affected coronary arteries (14.2 ± 8.6% vs. 9.5 ± 7.0%; *p* = 0.015). LGE volume regression in the MI culprit coronary artery regions was observed in 19 (70%) patients. A small proportion (*N* = 3, 10%) of patients with NSTEMI exhibited MVO on CMR.

**Table 4:** Left ventricle tissue characterization in NSTEMI patients at baseline and follow-up and in control subjects.

Global left ventricle characteristics	MI patients index event (*N* = 30)	MI patients follow-up (*N* = 27)	*p*-Value	Control subjects (*N* = 10)	*p*-value^†^
Native T1, ms	1,053 ± 52	1,026 ± 42	0.333	1,024 ± 15	0.198
Post-contrast T1, ms	328 ± 62	344 ± 58	0.198	314 ± 37	0.167
Late gadolinium enhancement, %	9.4 ± 4.4	6.7 ± 4.4	0.051	–	–
- Basal segments, %	9.1 ± 5.1	5.6 ± 3.6	0.023		
- Mid-cavity segments, %	8.4 ± 2.9	7.1 ± 6.6	0.333		
- Apical segments, %	17.0 ± 7.7	7.7 ± 8.1	0.001		
Extracellular volume, %	26.0 ± 4.7	25.8 ± 3.3	0.310	21.4 ± 1.2	0.018
MI culprit coronary artery heart segments					
Native T1, ms	1,080 ± 89	1,025 ± 62	0.013	–	–
Post-contrast T1, ms	321 ± 65	340 ± 62	0.270	–	–
Late gadolinium enhancement, %	13.5 ± 6.8	9.5 ± 5.0	0.015	–	–
Extracellular volume, %	26.4 ± 4.9	26.9 ± 4.8	0.223	–	–
MI non-culprit coronary artery heart segments					
Native T1, ms	1,042 ± 58	1,028 ± 39	0.175	–	–
Post-contrast T1, ms	331 ± 61	346 ± 58	0.188	–	–
Late gadolinium enhancement, %	6.4 ± 3.4	5.5 ± 3.4	0.212	–	–
Extracellular volume, %	24.8 ± 4.6	24.7 ± 3.3	0.613	–	–

MI, myocardial infarction.

^†^ MI index event compared to control subjects.

**Figure 3:**
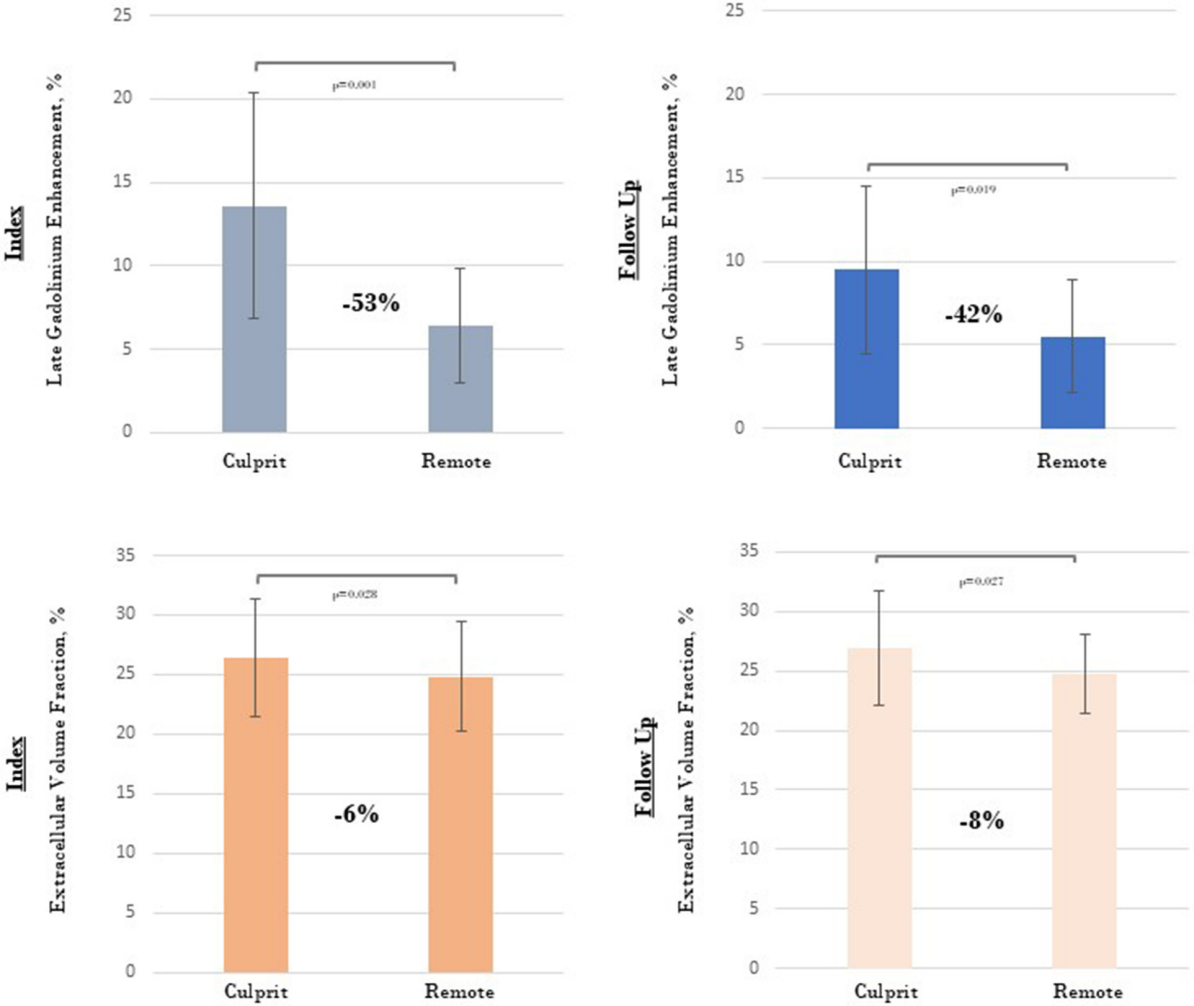
Evaluation of late gadolinium enhancement and extracellular volume fraction measurements in areas supplied by the myocardial infarction culprit and non-culprit coronary arteries.

Patients with NSTEMI had a higher ECV than the control subjects (26.0 ± 4.7% vs. 21.4 ± 1.2%; *p* = 0.018). However, ECV measurements did not change in the MI culprit coronary artery heart territories or the remote myocardium over time. (Table 4) Significant differences in LGE and ECV measurements were observed between the heart regions supplied by the MI culprit and the remote myocardium. (Figure 3) Patients with single- and multivessel CAD presented comparable LGE and ECV measurements in non-infarct-related regions of the heart.

The myocardial tissue characteristics of heart segments affected by MI-related coronary arteries are shown in Table 5 according to the CFR and IMR values at the initial and follow-up assessments. No correlation was found between invasive functional measurements and native T1, LGE, or ECV in the initial or subsequent cardiac imaging studies. Abnormal baseline CFR measurements (<2.0) and extensive microcirculatory dysfunction (IMR > 40) were not predictive of higher LGE or ECV measurements at baseline or during the follow-up.

**Table 5:** Left ventricle tissue characterization in the heart regions supplied by the MI culprit coronary artery, according to invasive CFR and IMR measurements at baseline and follow-up.

CMR tissue characteristics	MI patients index event (*N* = 30)			*p*-value	MI patients follow-up (*N* = 27)			*p*-value
	CFR < 2.0 vs. ≥ 2.0		IMR ≤ 40 vs. > 40		CFR < 2.0 vs. ≥ 2.0		IMR ≤ 40 vs. > 40	
No. of patients	18 (60%) vs. 12 (40%)		25 (83%) vs. 5 (17%)		7 (26%) vs. 20 (74%)		26 (96%) vs. 1 (4%)	
Native T1, ms	1,049 ± 88 vs. 1,102 ± 97		1,062 ± 99 vs. 1,081 ± 40	0.741	1,094 ± 98 vs. 1,080 ± 60		1,084 ± 85 vs. 1,103	0.196
Late gadolinium enhancement, %	12.9 ± 8.7 vs. 14.1 ± 9.7		12.9 ± 9.2 vs. 16.1 ± 5.7	0.323	15.5 ± 8.6 vs. 12.0 ± 8.3		12.8 ± 8.3 vs. 22.6	0.304
Extracellular volume, %	25.4 ± 4.2 vs. 29.7 ± 5.9		26.3 ± 5.3 vs. 27.8 ± 2.6	0.278	27.8 ± 4.7 vs. 27.6 ± 5.2		27.6 ± 5.0 vs. 28.4	0.872
CMR tissue characteristics	CFR ≥ 2.0 IMR ≤ 40	CFR < 2.0 IMR ≤ 40	CFR < 2.0 IMR > 40		CFR ≥ 2.0 IMR ≤ 40	CFR < 2.0 IMR ≤ 40	CFR < 2.0 IMR > 40	
No. of patients	12 (40%)	13 (43%)	5 (17%)		20 (74%)	6 (22%)	1 (4%)	
Native T1, ms	1,101 ± 97	1,044 ± 93	1,081 ± 20	0.346**†**	1,030 ± 60	1,037 ± 31	1,048	0.914^†^
Late gadolinium enhancement, %	14.1 ± 9.7	12.8 ± 9.1	16.1 ± 5.7	0.210**†**	6.6 ± 6.8	9.4 ± 7.0	14.6	0.333^†^
Extracellular volume, %	29.7 ± 5.9	24.6 ± 4.2	27.8 ± 2.6	0.198**†**	25.1 ± 4.3	30.5 ± 4.5	24.8	0.310**†**

CFR, coronary flow reserve; IMR, index of microcirculatory resistance.

^†^ differences between CFR/IMR groups.

During hospitalization, peak troponin levels correlated with the volume of LGE in the MI culprit coronary artery at the initial assessment (*r* = 0.535, *p* = 0.005). However, LGE volume regression over time in the MI culprit coronary artery regions was not associated with factors such as age, sex, prior MI, peak troponin level, multivessel coronary disease, CFR (≥2.0), or IMR ≤ 40 measurements.

### Reproducibility of LGE and ECV mapping values

Regarding the initial CMR procedure, global LGE mapping demonstrated high intra- and inter-observer reproducibility, with an intraclass correlation coefficient (ICC) of 0.92 (95% CI 0.83; 0.96) and 0.90 (95% CI 0.80; 0.95), respectively. Global ECV mapping exhibited low intra- and inter-observer variability with an ICC of 0.89 (95% CI 0.81; 0.95) and 0.88 (95% CI 0.79; 0.94). In the follow-up cardiac scans, global LGE mapping presented good intra- and inter-observer reproducibility, with ICC of 0.89 (95% CI 0.80; 0.95) and 0.88 (95% CI 0.80; 0.93), respectively. Moreover, global ECV mapping showed high intra-observer reproducibility with an ICC of 0.90 (95% CI 0.80; 0.96) and low inter-observer variability with an ICC of 0.88 (95% CI 0.78; 0.93) (Bland-Altman plots, Supplementary Material).

## Discussion

This study evaluated the temporal changes in LGE and ECV using CMR imaging in patients with type 1 NSTEMI at the index ischemic event and during the follow-up period. We assessed localized myocardial fibrosis using LGE, whereas diffuse fibrosis was quantified using ECV in regions supplied by both MI culprit and non-culprit coronary arteries. In contrast to most studies on post-MI CMR assessment, which have predominantly focused on STEMI, our study characterized myocardial fibrosis in patients with NSTEMI. Our results suggest that the development of myocardial fibrosis in NSTEMI is predominantly focal and localized in the cardiac regions supplied by the MI-affected coronary arteries. Diffuse myocardial fibrosis did not change over time. Despite the high prevalence of multivessel coronary artery disease (53%), significant changes in either focal or diffuse myocardial fibrosis were not observed in the remote regions of the heart after NSTEMI (Table 4).

NSTEMI, the predominant form of ACS, exhibits diverse pathophysiological mechanisms and varying risk levels for mortality and MACE (7). Although it demonstrates lower immediate hospital mortality than STEMI, NSTEMI presents a worse long-term prognosis and a higher prevalence of multivessel CAD (21). In type 1 NSTEMI, the rupture or erosion of an atherothrombotic plaque occurs within the affected coronary artery. This condition typically manifests as a non-occlusive thrombus in the culprit coronary artery and results in subendocardial injury/necrosis of the heart tissue (10). Our study protocol employed multiple criteria to assess the mechanisms of MI and enhance consistency in enrolling patients with type 1 NSTEMI.

In the late 1970s, Reimer and Jennings conducted experiments on dogs that revealed the progression of necrosis. Their findings showed that cell death advanced as a “ wavefront,” progressing from the subendocardium to the epicardium. Furthermore, histological examination of the infarct border revealed viable myocardium islands within necrotic areas and necrosis within viable tissue regions (22). T1-weighted LGE has been established as a surrogate for infarct size and myocardial viability (16) due to its close correlation with histopathologically proven myocardial necrosis (23). More recent CMR parametric (mapping) techniques that assess changes in T1, LGE, and ECV within infarcted regions and the remote myocardium offer a potentially more objective approach and facilitate operator-independent heart tissue characterization (16).

Loss of contractility after acute MI alters heart-loading conditions and triggers neurohormonal compensatory mechanisms, representing an adaptive response to maintain cardiac output despite myocardial tissue loss. This process may result in adverse changes in LV structure and geometry (remodeling), including thinning of the infarcted myocardium, LV dilatation, and eccentric hypertrophy of the remote healthy myocardium, leading to an increase in LV volume and overall contractility dysfunction over time (24). CMR studies conducted post-MI have primarily focused on predicting LV remodeling in patients with STEMI. This group tended to exhibit more LV uniformity alterations than NSTEMI, likely due to greater myocardial damage (24). Additionally, the intensity of the inflammatory response triggered by MI is proportional to the extent of ischemia and is, therefore, typically higher in STEMI than in NSTEMI (25). Our study cohort exhibited a substantial elevation in peak troponin levels (Table 1) during the index ischemic event, which was related to the LGE volume in the MI culprit coronary artery regions. However, significant LV remodeling was not observed, as evidenced by the stability of mass, volume, and function over time in patients with NSTEMI.

Cellular edema triggered by ischemia can be identified through native T1 mapping in both STEMI and NSTEMI patients (4), and is at least as sensitive as T2-STIR, particularly for patients with smaller infarcts (26). However, no specific threshold value can be universally applied to individual patients due to the inherent variability of native T1 values among individuals and the influence of field strength and infarct size on T1 values (26). Furthermore, Carrick et al. (27) observed that left ventricles that went on to remodel following STEMI had significantly higher native T1 in their remote myocardium during their early scans and attributed this feature to edema. In our cohort, native T1 mapping showed significantly longer T1 relaxation times at baseline than at follow-up (*p* = 0.013) in the MI culprit coronary artery regions of the heart, as edema was more pronounced in the infarcted area in the early MI stages. Moreover, our patients with NSTEMI did not exhibit significant alterations in native T1 values within the remote myocardium. This finding contrasts with that of a previous study focused on reperfused STEMI, which demonstrated that native T1 measurements in areas remote from the infarct site were independently associated with unfavourable LV remodeling and MACE (28).

Myocardial infarction size, a critical determinant of post-MI morbidity and mortality, represents the area of irreversibly damaged myocardium. CMR imaging with LGE is considered the gold standard for quantifying infarct size, demonstrating superior performance compared to other imaging methods in detecting subendocardial or previously unrecognized smaller infarcts (29). In the early stages of MI, the interstitial volume expands due to cardiomyocyte rupture, and LGE reflects both cellular necrosis and expanded extracellular volume in the acutely edematous myocardium. (Figure 2) At later post-MI stages, LGE uptake indicates replacement fibrosis. As such, infarct size can vary significantly at different imaging time points, related to ischemia/reperfusion injury, tissue healing, and the extent of irreversible tissue damage (i.e., subendocardial, transmural, or patchy), which should be taken into consideration when interpreting LGE imaging (4, 16). These findings have been previously reported by Dall'Armellina et al., showing a significant reduction in LGE from 24 h to 6 months in 30 patients with reperfused STEMI (30). Similarly, our NSTEMI cohort exhibited decreased LGE at follow-up, particularly in the basal and apical heart segments, and only significantly in the regions supplied by the MI culprit coronary artery (*p* = 0.015). (Table 4) Peak troponin levels at index hospitalization correlated with LGE volume in the affected coronary artery, which appeared to indicate consistent recruitment of type 1 NSTEMI in our cohort and adequate identification of the MI culprit coronary artery. (Figure 3) Nevertheless, the reduction in myocardial fibrosis over time represents a potential mechanism for improved outcomes in patients following MI (5). Although our results indicate that a reduction in LGE volume within the affected coronary artery heart area was frequently observed (70%), focal myocardial fibrosis regression could not be predicted by factors such as age, prior MI, peak troponin levels, the presence of multivessel coronary disease, or impaired microvascular function (CFR and IMR). Despite the well-established relationship between infarct transmurality and LV remodeling, the precise mechanisms linking the subendocardial infarct size to LV shape changes in NSTEMI remains incompletely understood (25).

CMR with T1 mapping can assess diffuse fibrosis through ECV quantification, enabling the tracking of potential dynamic changes in the cell and matrix compartments post-MI (5). In infarcted heart tissue, ECV was a significant predictor of LV wall recovery after adjusting for myocardial LGE (31). In addition to the replacement of cardiomyocytes by the extracellular matrix in the infarcted myocardium, ECV is often acutely elevated in the non-infarcted regions of STEMI survivors (32). Higher remote ECV measurements correlate with unfavourable LV remodeling, contractility impairment, and increased LV volume (33). Compared to control subjects, our ischemic patients exhibited acutely elevated ECV measurements. However, unlike native T1 values, a significant reduction in the ECV was not detected over time. This discrepancy may be attributed to the fact that these two parameters capture different compartments. ECV captures the extracellular components of the myocardium (e.g., extracellular matrix and vascular spaces), whereas native T1 is a composite signal of both the cellular and extracellular compartments of the myocardium (32). Our findings suggest that in NSTEMI, reactive diffuse fibrosis develops in both the infarcted areas and remote myocardium. However, it appears to lack the same dynamic nature of collagen turnover as that previously observed in patients with STEMI, even among NSTEMI cases with multivessel CAD as documented in our study group (32, 33).

Our results showed that coronary flow (CFR) impairment was more common than elevated microcirculatory resistance (IMR). (Table 2) Furthermore, throughout the follow-up period, CFR measurements showed significant improvement in the MI-affected coronary artery, and the incidence of patients with an IMR > 40 was uncommon. Patients with CFR measurements <2.0 or IMR > 40 did not demonstrate a correlation with more extensive LGE or ECV in the cardiac regions supplied by the MI culprit coronary artery on either the initial or follow-up cardiac scans. Previous studies have demonstrated minimal or no correlation between infarct size as measured by CMR and IMR following STEMI (34, 35). Additionally, research on post-MI CMR imaging and microcirculation function assessment has seldom focused on patients with NSTEMI (36).

Further research is needed to optimize the clinical utility of CMR in post-MI patients, given its limited accessibility in clinical practice. Its role in NSTEMI remains insufficiently defined, particularly regarding how the myocardial injury pattern evolves and influences long-term outcomes. The heterogeneous pathophysiology and typically smaller infarct size in NSTEMI pose challenges for CMR tissue characterization. Nonetheless, CMR can identify high-risk features in patients with NSTEMI and preserved LVEF, such as extensive myocardial fibrosis and/or microvascular dysfunction, which may benefit from more comprehensive cardioprotective therapy or close monitoring. It can assess the extent of salvageable myocardium, thereby informing decisions regarding revascularization strategies. Additionally, it can assist in differentiating ischemic type 1 lesions from supply-demand mismatch damage observed in type 2 MI, which may have divergent prognostic implications. Ultimately, employing a precision medicine approach with CMR may refine risk stratification in NSTEMI, enabling advanced therapies to target patients with potentially reversible or modifiable myocardial injury.

### Study limitations

This investigation was conducted at a single center and included patients diagnosed with NSTEMI, a medical condition that can manifest from diverse underlying pathophysiological mechanisms. The study protocol implemented multiple criteria to ensure the consistent recruitment of patients with type 1 NSTEMI and accurate identification of the MI culprit coronary artery. Nevertheless, this methodology may have introduced substantial selection bias, as it required the inclusion of only patients with unequivocal evidence of acute plaque rupture or vessel occlusion. Our findings demonstrated an increased LGE volume in the myocardial regions supplied by the MI culprit coronary artery (infarct region). Moreover, significant differences were observed in LGE and ECV measurements between the MI culprit coronary artery areas and the remote myocardium (Figure 3), indicating adequate identification of the MI culprit coronary artery in the NSTEMI cohort. The small sample size in our study limits the generalizability of the findings and elevates the risk of type II errors, as reduced statistical power may impede the identification of potential relationships between T1 measures and clinical outcomes or invasive microvascular function data. The inclusion of individuals with a history of MI may have led to an overestimation of LGE in certain patients. Nonetheless, our study protocol randomly enrolled patients with NSTEMI and employed automated LGE and ECV mapping, facilitating an operator-independent assessment of temporal changes in myocardial fibrosis in both infarcted and remote areas of the myocardium. Although our study did not include longitudinal validation and considering that infarct size may significantly vary at different imaging time points, we consistently performed CMR procedures within the first 5–10 days following hospital discharge and subsequently more than six months after the initial hospitalization (IQR 7, 9), at which stage mature myocardial fibrosis is expected (28). In our study, the potential influence of medical treatment on myocardial remodeling was minimized, as most patients were exposed to therapies known to affect myocardial fibrosis (ACEi/ARA/ARNI, beta-blockers), and LVEF was not significantly compromised after MI **(**Table 1).

## Conclusions

After NSTEMI, LGE decreased in the heart regions supplied by the culprit coronary arteries. However, the ECV measurements remained unchanged. Multivessel CAD was not associated with significant changes in myocardial fibrosis in the remote myocardium.

## Data Availability

The raw data supporting the conclusions of this article will be made available by the authors, without undue reservation.

## Supplementary material

Supplementary Figure A Bland-Altman plot of intra-observer reproducibility in LGE mapping in the first CMR procedure

Supplementary Figure B Bland-Altman plot of inter-observer reproducibility in LGE mapping in the first CMR procedure

Supplementary Figure C Bland-Altman plot of intra-observer reproducibility in ECV mapping in the first CMR procedure

Supplementary Figure D Bland-Altman plot of inter-observer reproducibility in ECV mapping in the first CMR procedure

Supplementary Figure E Bland-Altman plot of intra-observer reproducibility in LGE mapping in the second CMR procedure

Supplementary Figure F Bland-Altman plot of inter-observer reproducibility in LGE mapping in the second CMR procedure

Supplementary Figure G Bland-Altman plot of intra-observer reproducibility in ECV mapping in the second CMR procedure

Supplementary Figure H Bland-Altman plot of inter-observer reproducibility in ECV mapping in the second CMR procedure Legend: CMR cardiac magnetic resonance; ECV extracellular volume fraction; LGE late-enhancement gadolinium *Dotted lines delineate the limits of agreement that include 95% of all values
