# Supplementary material for: Cardiac T1 mapping in non-ST-segment elevation myocardial infarction: temporal changes in myocardial fibrosis
Source: Front Cardiovasc Med. 2025 May 23;12:1563368. doi: 10.3389/fcvm.2025.1563368 (PMC12141208; doi:10.3389/fcvm.2025.1563368)
Supplement: Supplementary Figure H — Bland-Altman plot of inter-observer reproducibility in ECV mapping in the second CMR procedure Legend: CMR cardiac magnetic resonance; ECV extracellular volume fraction; LGE late-enhancement gadolinium *Dotted lines delineate the limits of agreement that include 95% of all values [file Datasheet1.pdf]

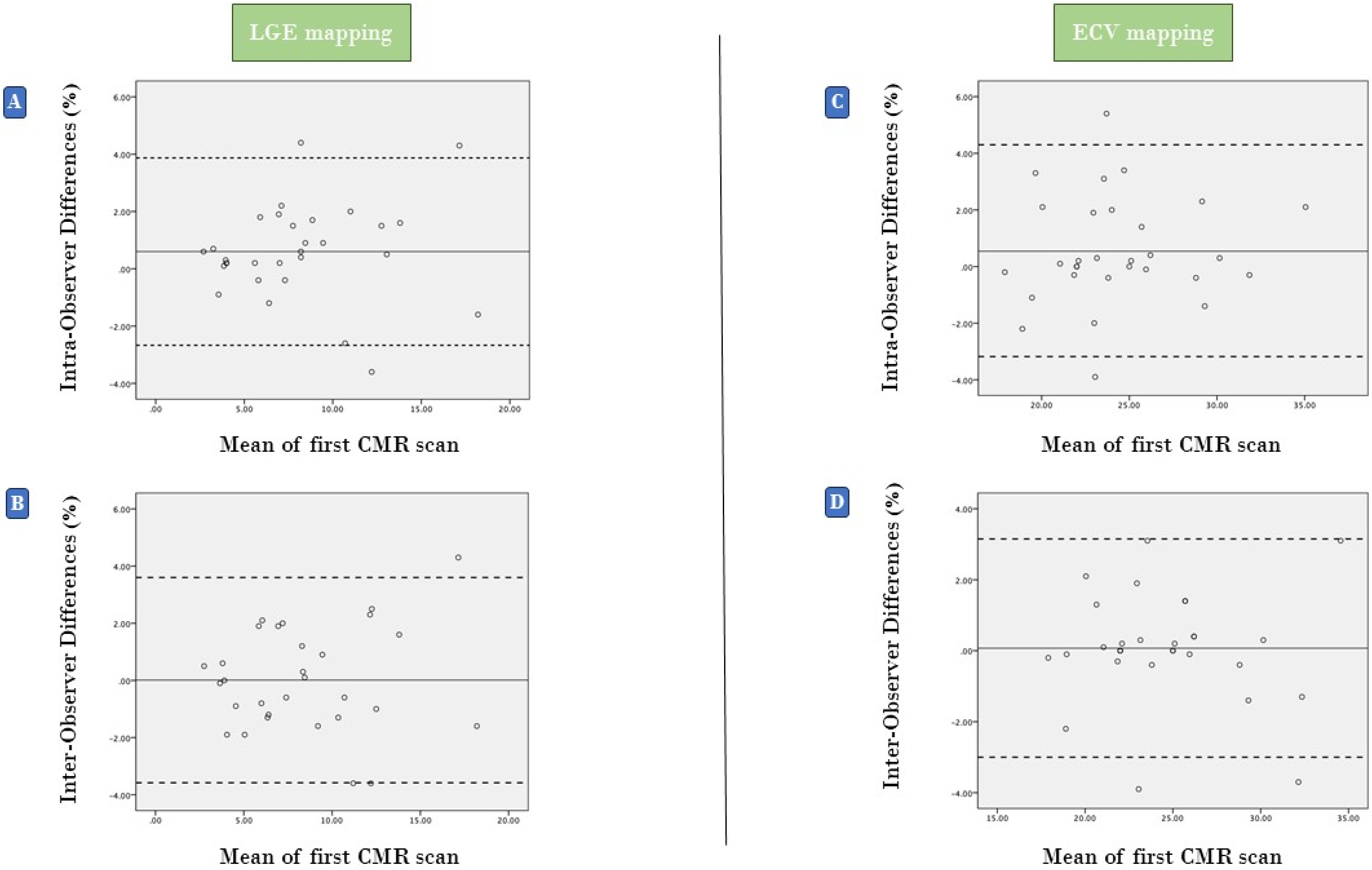
LGE mapping
ECV mapping
A
Intra-Observer Differences (%)
6.00
4.00
2.00
.00
-2.00
-4.00
.00
5.00
10.00
15.00
20.00
Mean of first CMR scan
B
Inter-Observer Differences (%)
Mean of first CMR scan
C
Intra-Observer Differences (%)
20.00
25.00
30.00
35.00
Mean of first CMR scan
D
Inter-Observer Differences (%)
15.00
Mean of first CMR scan

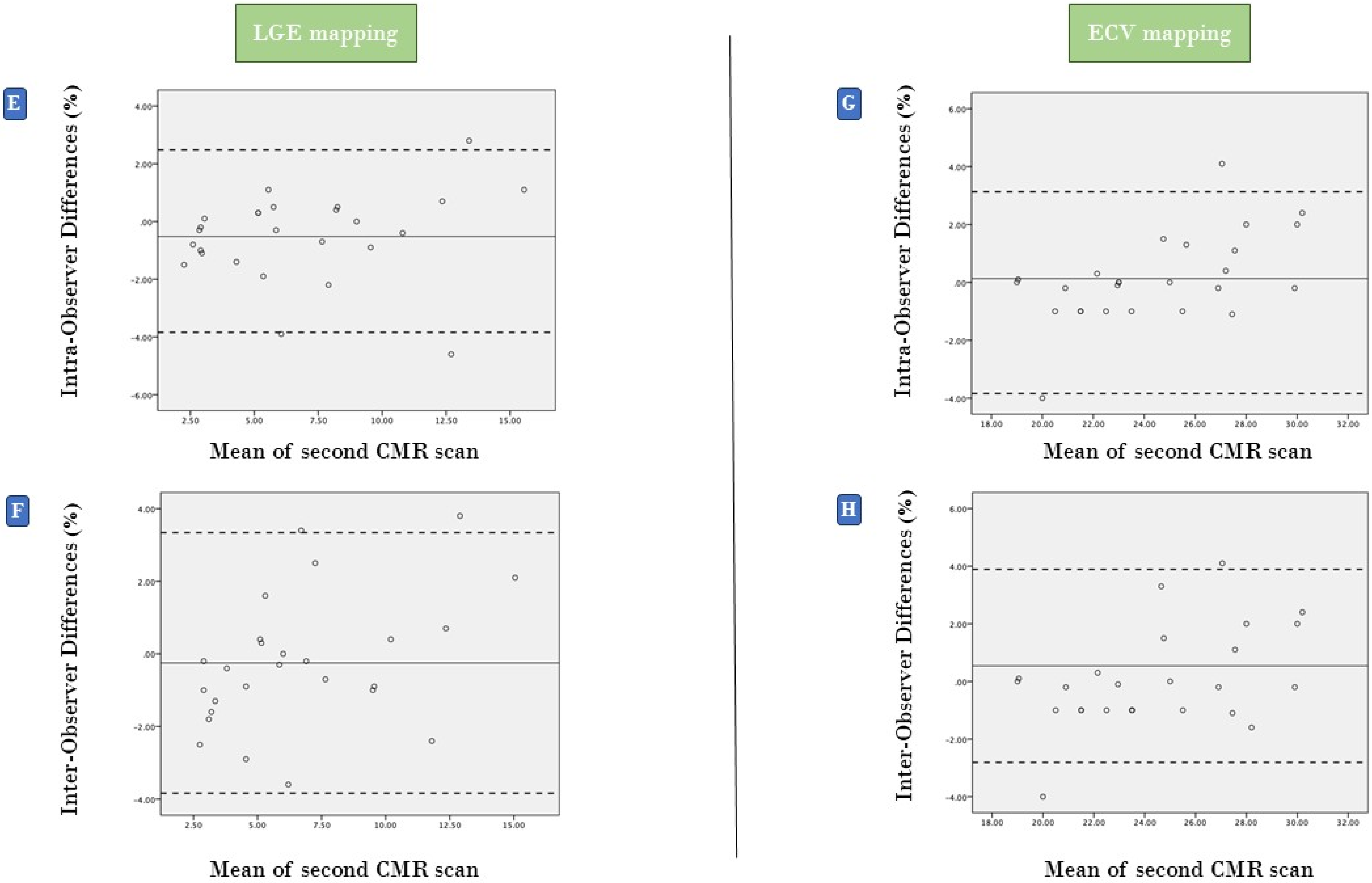
LGE mapping
ECV mapping
E
Intra-Observer Differences (%)
4.00
2.00
.00
-2.00
-4.00
-6.00
2.50
5.00
7.50
10.00
12.50
15.00
Mean of second CMR scan
F
Inter-Observer Differences (%)
4.00
2.00
.00
-2.00
-4.00
2.50
5.00
7.50
10.00
12.50
15.00
Mean of second CMR scan
G
Intra-Observer Differences (%)
6.00
4.00
2.00
.00
-2.00
-4.00
18.00
20.00
22.00
24.00
26.00
28.00
30.00
32.00
Mean of second CMR scan
H
Inter-Observer Differences (%)
6.00
4.00
2.00
.00
-2.00
-4.00
18.00
20.00
22.00
24.00
26.00
28.00
30.00
32.00
Mean of second CMR scan
